# The Wound-Healing Potential of *Olea europaea* L. Cv. Arbequina Leaves Extract: An Integrated In Vitro, In Silico, and In Vivo Investigation

**DOI:** 10.3390/metabo12090791

**Published:** 2022-08-25

**Authors:** Tarfah Al-Warhi, Abeer H. Elmaidomy, Sherif A. Maher, Dalia H. Abu-Baih, Samy Selim, Mha Albqmi, Mohammad M. Al-Sanea, Taghreed S. Alnusaire, Mohammed M. Ghoneim, Ehab M. Mostafa, Shaimaa Hussein, Ashraf K. El-Damasy, Entesar Ali Saber, Mahmoud A. Elrehany, Ahmed M. Sayed, Eman M. Othman, Mohamed El-Sherbiny, Usama Ramadan Abdelmohsen

**Affiliations:** 1 Department of Chemistry, College of Science, Princess Nourah bint Abdulrahman University, Riyadh 11671, Saudi Arabia; 2 Department of Pharmacognosy, Faculty of Pharmacy, Beni-Suef University, Beni-Suef 62511, Egypt; 3 Department of Biochemistry, Faculty of Pharmacy, Deraya University, New Minia 61111, Egypt; 4 Department of Clinical Laboratory Sciences, College of Applied Medical Sciences, Jouf University, Sakaka 72341, Saudi Arabia; 5 Chemistry Department, College of Science and Arts, Jouf University, Alqurayyat 77447, Saudi Arabia; 6 Pharmaceutical Chemistry Department, College of Pharmacy, Jouf University, Sakaka 72341, Saudi Arabia; 7 Biology Department, College of Science, Jouf University, Sakaka 72341, Saudi Arabia; 8 Pharmacognosy and Medicinal Plants Department, Faculty of Pharmacy (Boys), Al-Azhar University, Cairo 11884, Egypt; 9 Department of Pharmacy Practice, College of Pharmacy, Al Maarefa University, Ad Diriyah 13713, Saudi Arabia; 10 Department of Pharmacognosy, College of Pharmacy, Jouf University, Sakaka 72341, Saudi Arabia; 11 Department of Pharmacology, College of Pharmacy, Jouf University, Sakaka 72341, Saudi Arabia; 12 Department of Medicinal Chemistry, Faculty of Pharmacy, Mansoura University, Mansoura 35516, Egypt; 13 Brain Science Institute, Korea Institute of Science and Technology (KIST), Seoul 02792, Korea; 14 Department of Histology and Cell Biology, Faculty of Medicine, Minia University, Minia 61519, Egypt; 15 Department of Histology and Cell Biology, Deraya University, Universities Zone, New Minia 61111, Egypt; 16 Department of Pharmacognosy, Faculty of Pharmacy, Nahda University, Beni-Suef 62513, Egypt; 17 Department of Biochemistry, Faculty of Pharmacy, Minia University, Minia 61519, Egypt; 18 Department of Bioinformatics, Biocenter, University of Würzburg, Am Hubland, 97074 Wuerzburg, Germany; 19 Department of Basic Medical Sciences, College of Medicine, AlMaarefa University, Riyadh 11597, Saudi Arabia; 20 Department of Anatomy, Faculty of Medicine, Mansoura University, Mansoura 35516, Egypt; 21 Department of Pharmacognosy, Faculty of Pharmacy, Minia University, Minia 61519, Egypt; 22 Department of Pharmacognosy, Faculty of Pharmacy, Deraya University, New Minia 61111, Egypt

**Keywords:** *Olea*, olive, LC–HRESIMS, wound, *TNF*-*α*, *TGF*-*β*, *MMP-1*, virtual docking

## Abstract

*Olea europaea* L. Cv. Arbequina (OEA) (Oleaceae) is an olive variety species that has received little attention. Besides our previous work for the chemical profiling of OEA leaves using LC–HRESIMS, an additional 23 compounds are identified. An excision wound model is used to measure wound healing action. Wounds are provided with OEA (2% *w*/*v*) or MEBO^®^ cream (marketed treatment). The wound closure rate related to vehicle-treated wounds is significantly increased by OEA. Comparing to vehicle wound tissues, significant levels of *TGF-β* in OEA and MEBO^®^ (*p* < 0.05) are displayed by gene expression patterns, with the most significant levels in OEA-treated wounds. Proinflammatory *TNF-α* and *IL-1β* levels are substantially reduced in OEA-treated wounds. The capability of several lignan-related compounds to interact with *MMP-1* is revealed by extensive in silico investigation of the major OEA compounds (i.e., inverse docking, molecular dynamics simulation, and Δ*G* calculation), and their role in the wound-healing process is also characterized. The potential of OEA as a potent *MMP-1* inhibitor is shown in subsequent in vitro testing (IC_50_ = 88.0 ± 0.1 nM). In conclusion, OEA is introduced as an interesting therapeutic candidate that can effectively manage wound healing because of its anti-inflammatory and antioxidant properties.

## 1. Introduction

Worldwide, wounds pose serious health risks, placing significant financial, commercial, and communal stress on healthcare organizations, caregivers, patients, and families [1,2]. Wounds are reported as natural, thermal, chemical traumas, or abuses that destroy skin integrity [3]. A complex interaction of various cell types is an essential response to tissue damage. The initial constriction of blood vessels and aggregation of platelet are meant to break off bleeding. An inflow of inflammatory cells, beginning with neutrophils, follows, which discharges several mediators, including cytokines, to develop angiogenesis, thrombosis, and re-epithelialization. Additionally, the fibroblasts deposit extracellular factors and act as scaffolds [4]. The inflammatory stage is marked by hemostasis, chemotaxis, as well as enhanced vascular permeation, which diminishes further destruction, excludes cellular debris, heals the wound, and encourages cellular migration. The inflammatory stage usually lasts several days [5]. Granulation tissue development, re-epithelialization, and neovascularization define the proliferative phase. This period might persist for several weeks. As the wound grows, it gains maximal strength throughout the maturation and remodeling period [6]. One of the key objectives of the wound healing process is the regeneration of new connective tissue [7]. These restoration events occur by accumulating several collagen-dependent and noncollagenous-dependent molecules to supplement the healing process of the extracellular matrix (ECM), which is significant for providing the cellular microenvironment necessary for morphogenesis and growth.

Proteinases known as matrix metalloproteinases (MMPs) contribute to ECM breakdown [7,8,9]. MMP activities are perfectly adjusted by controlling homeostatic environments at different stages including transcriptional level, precursor zymogen induction, ECM interplay, and suppression by internal inhibitors [9,10,11]. Disorders such as arthritis, tumor, atherosclerosis, nephritis, fibrosis, aneurysms, and tissue lesions can take place owing to a loss of regulatory activity [12]. Several studies have reported that MMPs are over-expressed in wounds (e.g., *MMP-1–3*) [13]. Consequently, inhibition of the catalytic activity of these hydrolytic enzymes was associated with a better and faster wound-healing process (i.e., better collagen maturation and crosslinking) [14].

The benefit of medicinal plants in curing wounds at different stages is widespread in almost all conventional medical systems worldwide. Significant potential for enhancing and improving the quality of wound healing has been shown in several herbal-based remedies [15,16], based on *Curcuma longa* (L.) [17], *Centella asiatica* [18], *Sphagneticola trilobata* [19], *Aloe barbadensis* [20], *Azadirachta indica* [21], and *Chamomilla recutita* [22]. The use of Theacea plant bioactive components for wound healing is patented [23]. Additionally, another reported patented is new herbal components for treating wounds, which consisted of *Curcuma longa*, *Hamil tonia suaveolens*, *Glycyrrhiza glabara*, *Tipha angustifolia*, *Azadirachta indica*, and *Sesamum indicum* (Til) oil [24]. Moreover, herbal-based pharmaceutical remedies are used, such as moist exposed burn ointment (MEBO^®^), which is composed of several amino acids and other plant-based constituents [25].

Since antiquity, OEA has been grown primarily for oil production in Mediterranean lands. Recently, the positive effects of biophenols (e.g., verbascoside, oleuropein, hydroxytyrosol, and luteolin-7-*O-β*-glucopyranoside) isolated from olive for human benefits (e.g., antihypertensive [26], cholesterol-lowering [27], cardioprotective [28], anti-inflammatory, and as co-adjuvant for obesity [29]) have been carefully established [30].

*Olea europaea* L. Cv. Arbequina, a cultivar of olives, is an olive variety species that has received little attention. Arbequina has lately become one of the world’s most important olive cultivars, owing partly to extensive cultivation and “super-high-density” plantations [31]. Arbequina trees can fit a variety of climatic and soil conditions. Nonetheless, it flourishes in long, hot, and dry summers and grows best in alkaline soils. Nonetheless, it is frost-resistant and pest-resistant [32]. Almost 78 percent of olive oil is grown on *Arbequina* rootstock [33]. As a result, Arbequina olives have one of the highest oil contents and are largely utilized in the production of olive oil [34]. Publicly, *Olea europaea* oil and polyphenol contents are fully documented to reduce oxidative stress, stimulate wound healing, and minimize inflammation [35,36,37,38,39,40]. However, the oil and polyphenol contents are generally affected by climatic conditions during ripening and the degree of maturation, especially the Arbequina types [41,42].

In an attempt to compare the cultivar olive with the wild types in the content and activities concerning this field, our previous work estimated the potential of OEA leaves (cultivated in Egypt) as an internal wound healer against gastric ulcers in a rat model compared to wild *O. europaea* [43]. The results showed that the crude extract of the OEA cultivar was richer with polyphenolic content using LC–HRESIMS. The ulcer index of the rat model was significantly decreased, and the mucosa from the lesions was protected [43].

**Figure 1:**
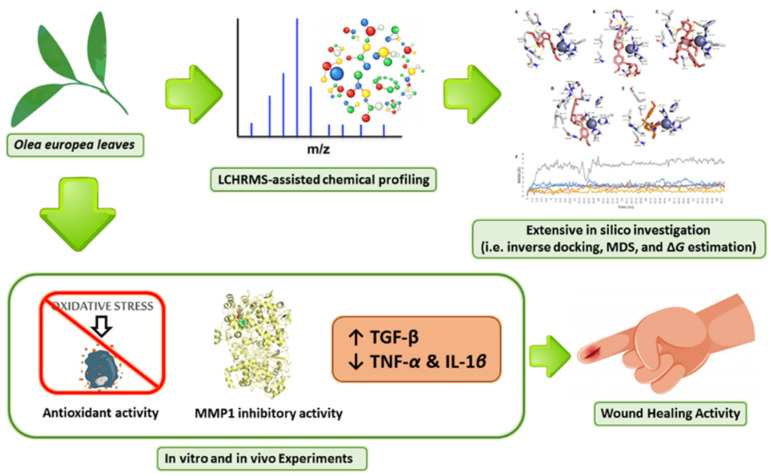
The general summary of the present research is as follows: *Olea europaea* leaves were extracted, chemical profiling was carried out, and finally the leaves were assessed for their potential wound healing activity by evaluating their antioxidant activity and ability to inhibit MMP1, upregulate *TGF-β* relative gene expression, and downregulate the relative gene expression of inflammatory cytokines (*TNF-α and IL-β1*).

To complement the previous work, we targeted the wound healing ability of OEA as an external wound healer by applying an excisional wound model. We focused on essential wound healing targets, encompassing transforming growth factor-beta (*TGF-β*), interleukin-1*β* (*IL-1β*) as well as tumor necrosis factor-α (*TNF-α*). Illustrating the mode of action of OEA using several in vitro and in silico assays. Additionally, we assessed the possible inhibitory effect of OEA on one of the key players in wound healing (i.e., *MMP-1*), as proposed by the in silico investigation. Figure 1 depicts the framework of the present investigation.

## 2. Materials and Methods

### 2.1. Plant Material

In April 2020, OEA leaves were harvested from Basita Farms in Aljouf, Saudi Arabia. Dr. Hamdan Ogreef (Camel and Range Research Center in Sakaka, Saudi, Arabia) graciously identified it. A voucher specimen (2020-BuPD 75) was kept at Beni-Suef University’s Pharmacognosy Department; Faculty-of-Pharmacy.

### 2.2. Chemicals and Reagents

All reagents and compounds were obtained from Sigma-Aldrich unless otherwise stated (Germany, Biosystems SA Costa Brava 30, Barcelona, Spain).

### 2.3. Plant Material Extraction

Two kilograms of OEA leaves were collected, meticulously cleaned, and air-dried for 10 days in the shade. The leaves were ground with an OC-60B/60B herb grinding mill (60–120 mesh, Henan, China—Mainland). The powdered leaves were soaked in a huge, closed glass jar for intensive extraction with 70% EtOH (15 L X3) and concentrated under reduced pressure at 45 °C. Following these steps, 80 grams of dried residue was obtained. It was saved at 4 °C for further phytochemical and biological screenings [44,45].

### 2.4. Metabolomic Analysis

According to our previously reported protocol [44], LC-HRMS-assisted metabolomic analysis of OEA was carried out. The detailed method is described in the supplementary file.

### 2.5. In Vivo Wound Healing Activity

#### 2.5.1. Animal Treatment

Eighteen wholesome adult male New Zealand Dutch breed albino rabbits participated in this investigation (2.1–2.7 kg). Polypropylene cages were used for animal housing. The normal pellet feed and unlimited water were freely available during the experiment. The seven days leading up to the experiment were spent acclimating the animals to the lab environment. Animals were introduced to a home with adequate ventilation under conditions of 25 ± 2 °C and relative moisture of 44–55% with 12 h cycles of dark/light.

#### 2.5.2. Samples Preparation for the Bioassay

In order to evaluate the effectiveness of OEA extract in healing wounds, excisional wound models were applied [42]. The extract was made for the wound models by dissolving OEA dry extract in carboxymethylcellulose (2 g of dried extract in 100 mL of 0.5% carboxymethylcellulose) and kept at 4 °C in the dark. Each test extract was applied topically to the wound site as soon as it was prepared [43].

#### 2.5.3. Model for Circular Excision Wounds

Excisional wounds were induced in rabbits [46,47]. In summary, 0.01 mL Ketalar^®^ (Ketalar, Sankyo Lifetech Co., Ltd., Tokyo, Japan) was used to anesthetize the animals. The back hairs of the rabbits were carefully shaved. The circular incision of each animal was made with a 6-mm biopsy punch by only excising the skin on the dorsal interscapular area. A sterile cotton swab soaked in 0.9% saline was used to clean the wounds. The induced wounds were left undressed throughout the whole duration of the study.

18 rabbits were divided to 3 groups. Each group comprised 6 rabbits. Group 1 (control rabbit): ulcers treated with vehicle only twice daily; Group 2: ulcers treated topically with OEA 2% *w*/*v* extract twice a day for 14 days until the wounds were completely cured; and Group 3: ulcers were topically treated with MEBO^®^ (Julphar Gulf Pharmaceutical Industries, Ras Al Khaimah, United Arab Emirates) (a comparable market product) twice daily for 14 days until the wounds were mostly cured.

#### 2.5.4. Collection of Tissue Samples

On days seven and fourteen, full-thickness skin biopsies of complete ulcers from each rabbit were taken under anesthesia. Tissue samples were sectioned into three halves. Most of the dissected wound tissue was utilized for gene expression research. For histological investigation, the remainder was kept in formalin [43].

#### 2.5.5. Percentage Wound Closure Rate

A camera (Fuji, S20 Pro, Sendai, Japan) was used to monitor the development of the wound region every three days until it healed fully. Using Image J 1.49 v software from the National Institutes of Health in Bethesda, Maryland, the wound area was assessed, and the wound closure rate was reported as a percentage change in the original wound area using the following formula:

$$Wound\;closure\;\left(\%\right)=\frac{Wound\;area\;on\;day\;0-Wound\;area\;on\;day\;nth}{Area\;of\;the\;wound\;on\;day\;0}\times 100$$

*n:* days numbers.

Additionally, a wound aspect ratio was established to explain observed variations in the shape and angular direction of wound contraction between groups. Using Image J, the length to width ratio was calculated from measurements of the wound’s length (measured from head to tail) and width.

#### 2.5.6. Histological Study

Dorsal skin samples from all wounds were taken and fixed in buffered formalin before being treated with a graded series of alcohol and xylene and subsequently immersed in paraffin blocks. Tissue slices were cut at a thickness of 4 µm and discolored with hematoxylin/eosin dye. The Leica Application Suite (Leica Microsystems, Wetzlar, Germany, a light microscope) was applied to evaluate and photograph the mounted slides [47].

#### 2.5.7. Gene Expression Analysis

##### Total RNA Extraction

In 0.5 mL TRIzol reagent (RNA Isolation Reagent, Invitrogen—ThermoFisher Products & Kits, Amresco, LLC-Solon, Waltham, MA, USA), 50 mg of dorsal skin tissue was homogenized using an ultrasonic homogenizer (Sonics-Vibracell, Sonics and Materials Inc., Newtown, Fairfield County, CT, USA). According to the guidelines of the producer, total RNA was extracted from dorsal skin tissues, and the concentration of RNA yield and purity were calculated [48].

##### Real-Time qRT–PCR

cDNA synthesis with a constant RNA concentration across all samples, the RevertAid H Minus First Strand cDNA synthesis kit was used as directed by the manufacturer (#K1632, Thermo Scientific Fermentas, St. Leon-Ro, Germany). SYBER Green (Thermo Scientific Fermentas St. Leon-Ro, Germany-Maxima SYBER Green qPCR Master Mix (2X)) was employed in real-time PCR using single-stranded cDNAs. A StepOne Real-Time PCR System (Applied Biosystems, Thermo Fischer Scientific, Waltham, MA, USA) was used to perform qRT–PCR. The set of primers used for real-time PCR is mentioned in Table 1 qRT–PCR was performed using 0.02 g RNA per reaction and 10 Pmol of particular primers for thirty cycles of 95 °C for ten seconds and 60 °C for one minute. After normalization to glyceraldehyde-3-phosphate dehydrogenase (GAPDH) as a housekeeping gene, gene expression levels were obtained. To assess the relative quantities of RNA, the comparative Ct approach was utilized. Formula 2 ^(−ΔΔCt)^ was used to determine the relative expression [12].

### 2.6. Molecular Modeling

#### 2.6.1. Prediction of the Molecular Targets of the Dereplicated Metabolites of OEA

By carrying out docking against all protein structures saved in the PDB (https://www.rcsb.org/) (accessed on 1 March 2022), several suggested targets for the OEA-isolated molecules were putatively determined. In this analysis, small overlapping grids are adaptively built to constrain the searching space on protein surfaces, allowing them to run many accurate dockings runs in a shorter time [49]. The information was compiled as a ranking of binding affinity scores. We utilized a binding affinity score threshold of 7 kcal/mol to recognize the ideal receptors for each OEA-isolated molecule.

#### 2.6.2. Molecular Dynamic Simulation

Molecular dynamics simulation and estimate of binding free energy were carried out as previously mentioned using Desmon software [50]. The complete technique is included in the supplemental file.

### 2.7. In Vitro MMP-1 Activity Assay

OEA was tested for its inhibitory activity against *MMP-1*. The enzyme inhibition assay was conducted in accordance with the company instructions (Abcam, Waltham, MA USA, Cat. No: ab118973).

### 2.8. Statistical Analyses

Data are reported as the mean ± standard deviation of the mean (*n* = 6). Tukey’s test for multiple comparisons was applied after one-way analysis of variance (ANOVA). Calculations involving statistics were performed using Graph Pad Prism 8 (San Diego, CA, United States). The outcomes were deemed significant when the *p* value was less than 0.05 [12].

## 3. Results

### 3.1. Chemical Dereplication of OEA Leaves Extract

According to our previous study, OEA crude extract dereplicated 18 metabolites using LC–HRESIMS, which identified as 3-hydroxy-12-oleanen-28-oic acid; 2,3-dihydroxy-13(18)-oleanen-28-oic acid; oleuropein; 2-(3,4-dihydroxyphenyl)ethanol; oliverixanthone; cleroindicin F; oleuropein 3″-Me ether; oleoside; 11-octadecen-9-ynoic acid; 3-hydroxy-12-ursen-28-oic acid, 3-ketone; 8-epimer, (3,4-dihydroxyphenylethyl) ester; chebulic acid 4,5-didehydro(E-), tri-Et ester; verbascoside; luteolin; olenoside A; olivine; olivacene; and oleacein [43].

**Figure 2:**
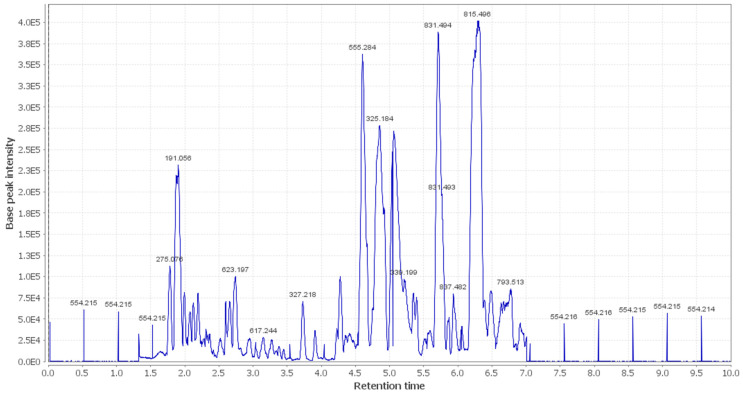
LC-HRESIMS chromatogram of the dereplicated metabolites of *OEA* (positive mode).

**Figure 3:**
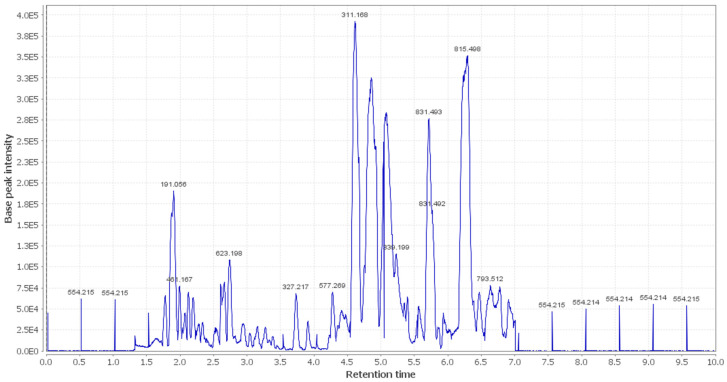
LC-HRESIMS chromatogram of the dereplicated metabolites of *OEA* (negative mode).

**Figure 4:**
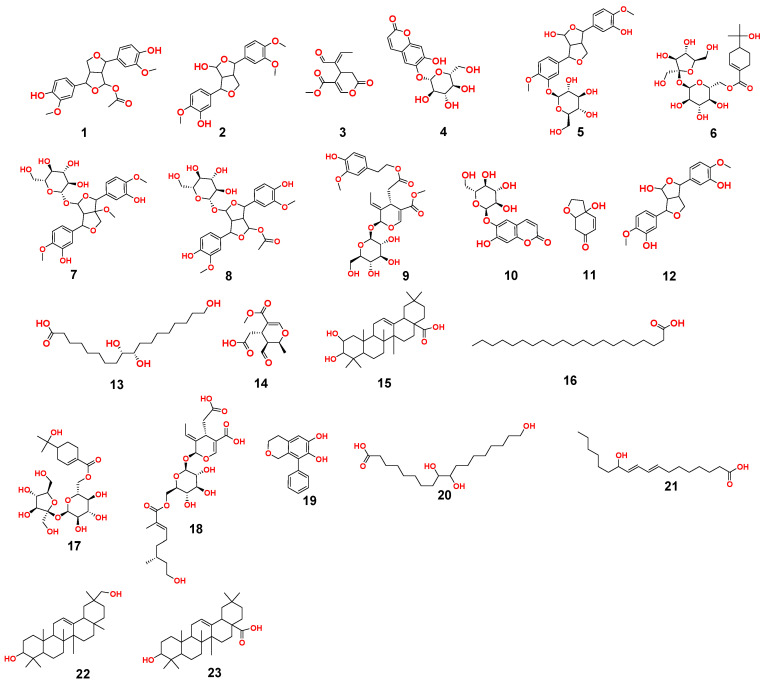
Dereplicated metabolites from LC-HRESIMS analysis of *OEA* (**1**–**23**).

In the present study, additional hits were introduced (Table 2, Figure 2, Figure 3 and Figure 4). The *m*/*z* 375.1444 and 389.1600. Mass ion peaks corresponded to the proposed molecular formulas C_20_H_22_O_7_ and C_21_H_24_O_7_ [M + H]^+^, which fit tertahydrofurofuran lignan 7,9′:7′,9-diepoxy-8,8′-lignan-3,3′,4,4′,8-pentol;3,3′-di-Me ether **1**, 7,9′:7′,9-diepoxy-8,8′-lignan-3,3′,4,4′,8-pentol; 3,3′,4′-tri-Me ether **2**, which was formerly extracted from *OEA* [35,36,37].

The *m*/*z* 223.06055 [M − H]^+^ and 341.08660 [M + H]^+^ molecular ion mass peaks, respectively, were characterized for the predicted molecular formulas C_11_H_12_O_5_ and C_15_H_16_O_9_, and indicated *S*-(*E*)-elenolide **3** and benzopyrene, 6,7-dihydroxy-2*H*-1-benzopyran-2-one;6-*O-β*-d-glucopyranoside **4**, respectively, which were formerly separated from *OEA* [35,38,39]. The ion mass peaks 537.1972, 509.22142 *m*/*z*, [M + H]^+^ for C_26_H_32_O_12_, C_22_H_36_O_13_ predicted molecular formulas, indicated the tertahydrofurofuran lignan nucleus of 7,9′:7′,9-diepoxy-8,8′-lignan-3,3′,4,4′,8-pentol, 3,3′-Di-Me ether,4-*O-β*-d-glucopyranoside **5**, which was divided from *OEA* [39], and 6-*O*-oleuropeoyl-sucrose **6**, which was divided from *OEA* [51]. Two ion peaks, with *m*/*z* 567.2078 and 579.20783 [M + H]^+^, predicted for molecular formulas C_27_H_34_O_13_ and C_28_H_34_O_13_ were dereplicated as 7,9′:7′,9-diepoxy-8,8′-lignan-3,3′,4,4′,5,8-hexol, 3,3′,5-tri-Me ether,8-*O-β*-d-glucopyranoside **7**, and 3,3′,4,4′,8-pentahydroxy-7,9′:7′,9-diepoxylignan,3,3′-di-Me ether, 8-Ac,4-*O-β*-d-glucopyranoside **8**, respectively, which were separated previously from *OEA* [37]. The mass ion peak at *m*/*z* 541.1921 reflected the predicted molecular formula C_25_H_32_O_13_ and indicated secoiridoid oleuropein **9**, which was divided from *OEA* [52]. Additionally, *m*/*z* 417.15389 and 155.0708, corresponding to the suggested formulas C_22_H_24_O_8_, C_8_H_10_O_3_ [M + H]^+^, which fit tertahydrofurofuran lignan 3,3′,4,4′,8-pentahydroxy-7,9′:7′,9-diepoxylignan; 3,3′-Di-Me ether,8-Ac **10**, which was already divided from *OEA* [35,36,37], and megaritolactones, halleridone **11**, which was also isolated from *OEA* [38,39].

Additionally, the *m*/*z* 341.0873, 331.24803, 243.0869, and 473.36213 analogous to the proposed molecular formulas C_15_H_16_O_9_ [M + H]^+^, C_18_H_36_O_5_ [M − H]^+^, C_11_H_14_O_6_ [M + H]^+^, and C_30_H_48_O_4_ [M + H]^+^ fit benzopyrene, fatty acid, triterpene compounds 6,7-dihydroxy-2*H*-1-benzopyran-2-one;6-*O-β*-d-glucopyranoside **12**, which was isolated from *OEA* [35], 9,10,18-trihydroxyoctadecanoic acid **13**, elenaic acid **14**, which were isolated from OEA [53,54,55,56,57], and 2,3-dihydroxy-13(18)-oleanen-28-oic acid **15**, respectively, which were previously isolated from *OEA* [58]. The *m*/*z* 277.2167 suggested the molecular formula C_18_H_30_O_2_ [M-H]^+^, fit fatty acids 11-octadecen-9-ynoic acid **16**, formally isolated from *OEA* [56,57]. The ion mass peaks at *m*/*z* 509.22142, 557.2229 [M − H]^+^, for C_22_H_36_O_13_, C_26_H_38_O_13_ indicated 6-*O*-oleuropeoyl-sucrose **17**, oleoside; 6′-*O*-(8-hydroxy-2,6-dimethyl-2E-octenoyl) **18**, which was isolated from *OEA* [51]. Moreover, *m*/*z* 243.1013, 331.2490, 295.2280, 443.3880, and 457.3682 [M + H]^+^, [M − H]+, for the predicted molecular formulas C_15_H_15_O_3_, C_18_H_35_O_5_, C_18_H_32_O_3_, C_30_H_50_O_2_, and C_30_H_49_O_3_ indicated benzopyran, fatty acid, and triterpenes, 3,4-dihydro-1-phenyl-1*H*-2-benzopyran-6,7-diol **19**, 9,10,18-trihydroxyoctadecanoic acid **20**, 12-hydroxy-8,10-octadecadienoic acid **21**, 12-oleanene-3,16-diol or 12-oleanene-3,28-diol or 13 (18)-oleanene-3,16-diol or 12-ursene-3,16-diol or 12-oleanene-3,28-diol **22**, and oleanolic acid **23**, respectively, were previously isolated from *OEA* [58].

### 3.2. Wound Healing 

#### 3.2.1. Wound Closure Rate

**Figure 5:**
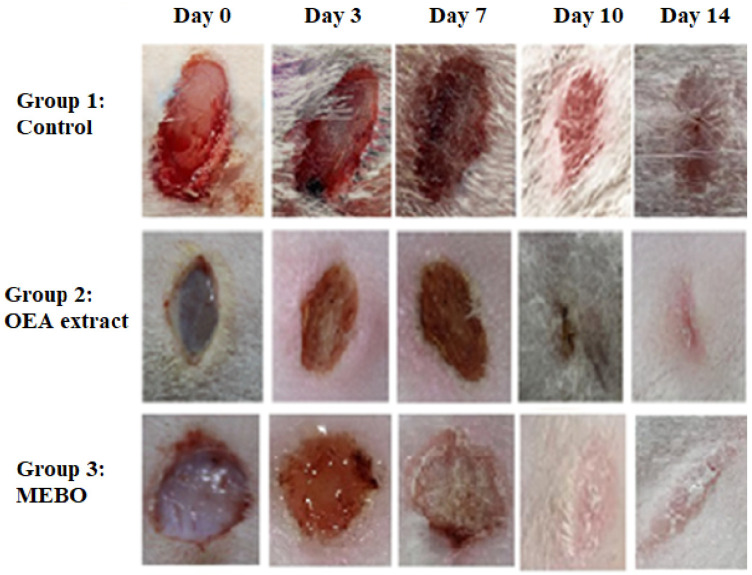
Wound closure rates were calculated using Image J software at various points (0, 3, 7, 10, and 14 days) after injury in each of the three groups (*n* = 6).

**Figure 6:**
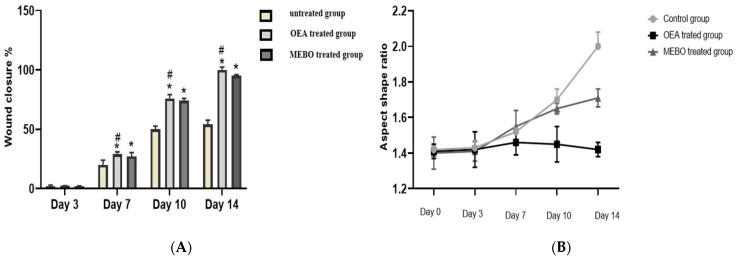
(**A**) Percentages of wound closure in all groups of the study (*n* = 6) with time after injury (0, 3, 7, 10, and 14 days). A two-way ANOVA test was performed to evaluate whether there was a significant difference between the groups. The data is presented as the mean ± SD. * *p* < 0.05 compared to the untreated group on the same day, and # *p* < 0.05 compared to the MEBO® group on the same day. (**B**) To clarify observed differences in the shape and direction of wound contraction between groups, the wound aspect ratio was calculated (length: width).

In the in vivo model, the rate of wound closure was improved in all studied groups in a time-dependent manner. On the third post-injury day, the rate of wound closure was approximately 10% in all groups, with the control group having the lowest and the treated group having the highest. There was no significant variation among groups (*p* < 0.05). On 7th and 10th post-excision days, the rates of wound closure in the OEA-treated group were 32% and 81%, respectively, which seemed to be statistically greater (*p* < 0.05) than those in the control group. The OEA group had faster wound closure than the MEBO^®^-treated group on the seventh and tenth days postinjury. On the fourteenth post-excision day, the rabbits treated with OEA were completely recovered from their wounds, and the wound contraction reached 100% in the OEA group (Figure 5 and Figure 6A,B).

#### 3.2.2. Histopathological Study

##### Seven Days Post-Treatment

- Group I (control group).

**Figure 7:**
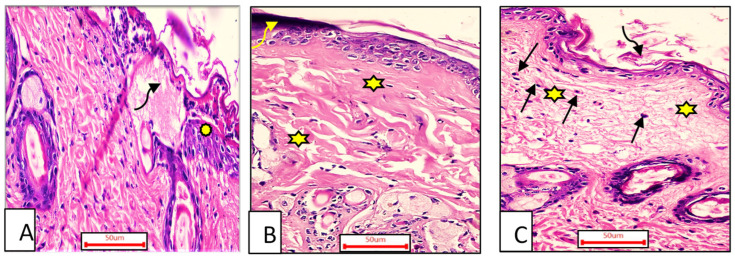
A sample of the wounded skin of individuals from each treatment group seven days after incision; Group I (**A**) showing the typical wound margin with the normal epidermis (asterisk). The wound was clogged with blood clots and granulation tissue (curved arrow). Group II (**B**) blood clot seen above the incision visible (curved arrow). Compactly arranged dermal collagen fibers are seen (aster). Group III (**C**) scar tissue (curved arrow) blocked the incision, and collagen fibers (stars) resembled the neighboring normal dermis with inflammatory cellular activity, particularly macrophages (black arrows). (H & E stain ×200).

The ordinary edge of the injury had a natural epidermis, normal hair follicles, well-formed collagen bundle dermis, and healthy sebaceous glands. Alternatively, the injury was packed with blood clots, inflammatory cellular infiltration, extravasated RBCs, and sloughed granulation material, with collagen tissues compactly organized in a strange pattern. The striated muscle appeared to have necrotic myo-fibers in the deepest area. (Figure 7A).

- Group II (OEA-treated).

The blood clot seen above the incision remained visible, with inadequate reepithelization, and the granulation tissue filling the defect from below was mostly cellular. In comparison to other treated groups, the heavy collagen was combined with fibers that were compactly formed in an irregular manner, leading to apparent scarring (Figure 7B).

- Group III (MEBO^®^-treated).

Epidermal cells can be seen spreading around the edges of the incision. The re-epithelization was lacking, though. Approximately mirroring the nearby normal dermis, collagen fibers and inflammatory cells infiltration were seen populating the damage with space between them. The reticular dermis had a large number of active extended spindle-shaped fibroblasts, basophilic cytoplasm, and open-face oval nuclei. (Figure 7C).

##### Fourteen Days after Treatment

- Group I (control group).

**Figure 8:**
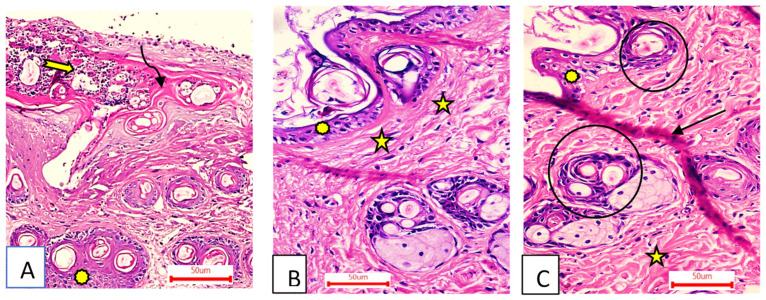
Group I (**A**) had a wide wound area (curved arrow), heavy inflammatory cellular infiltration in an acidophilic matrix (tailed arrow), and normal skin (asterisk). Group II (**B**) had an epidermis formed of 1–3 rows of epithelial cells (asterisk) and collagen fibers compactly arranged with prominent fibroblasts (stars). Group III (**C**) had typical epithelium, thin scar tissue extending into the dermis (black arrow), and reticular dermis with coarse, wavy collagen bundles arranged in different directions (star). Newly formed hair follicles (circles) (H&E stain ×200).

The wound area was larger and heavily covered in granulation tissue, which was made up of several connective tissue layers in an acidophilic matrix and covered a significant amount of inflammatory cellular infiltration. The dermis has substantial neovascularization and was made of thin, disordered collagen (Figure 8A).

- Group II (OEA-treated).

The wound had become plugged by scar tissue that had shrunk. In the epidermis, only one to three rows of epithelial cells have recovered. The granulation tissue from below was primarily cellular and fibroblast packed. Collagen fibers were disorganized–dense and compactly oriented in the reticular layer (Figure 8B).

- Group III (MEBO^®^-treated).

The ordinary squamous keratinized epithelium was present in the skin tissue. The dermis was covered in thin scar tissue. There were numerous hair follicles, a dermal matrix with blood capillaries, and no inflammatory cellular infiltration. The collagen bundles of the papillary dermis were depicted as thin interweaving bundles, while the reticular dermis evolved into rough, wavy bundles that were established in diverse routes (Figure 8C).

#### 3.2.3. Effect of OEA Treatment on Relative Gene Expression of *TGF*-β in Excisional Wounds

**Figure 9:**
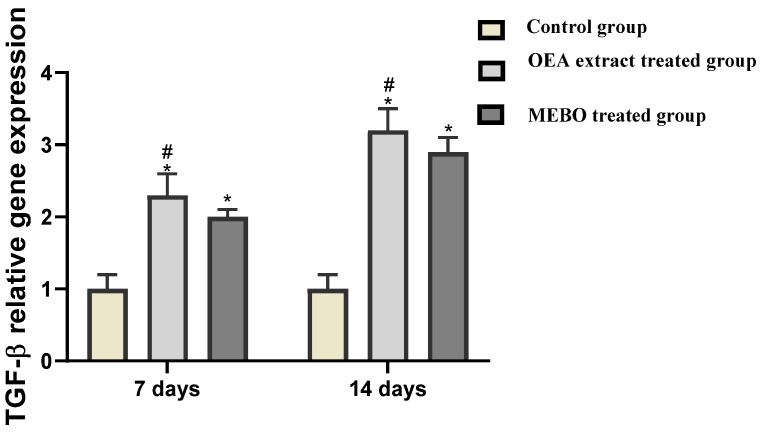
Gene expression in diverse categories of wounded tissues. qRT–PCR was used to examine gene expression in wound tissues. After being adjusted for GAPDH, the results displayed as fold change relative to the control group. The bars represent the mean ± SD. To determine whether there was a substantial difference between studied groups, a two-way ANOVA was performed, with * *p* < 0.05 comparing to the control group on a particular day and # *p* < 0.05 comparing to the MEBO^®^ group on the same day.

The relative expression expression of *TGF*-*β* in skin tissues was significantly statistically higher in OEA-treated injuries on the seventh and fourteenth days than in individuals from the control group (*p* < 0.05). Furthermore, OEA-treated wounds had considerable development in the expression of the marker compared to MEBO^®^-treated wounds (Figure 9).

#### 3.2.4. Effect of OEA on *IL-1β, TNF*-α and *MMP-1* Gene Expression in Excisional Injuries

**Figure 10:**
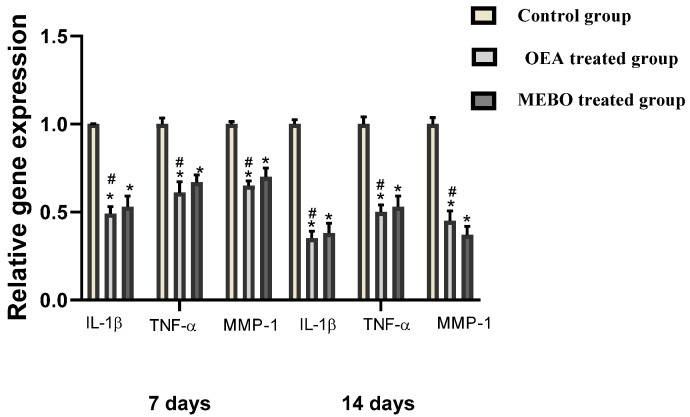
Gene expression in wounded rabbit tissues from several groups. qRT–PCR was utilized to assess gene expression in wounded tissues. After normalization to GAPDH, the data represented as fold change relative to the control group. The bars represent the mean ± SD. To determine whether there was a significant variation between studied groups, a one-way ANOVA test was utilized, with * *p* < 0.05 comparing to the control group on that day and # *p* < 0.05 comparing to the MEBO^®^ treated group on the same day.

According to the analysis of mRNA levels on the seventh postinjury day, inflammatory mediators (*IL-1β and TNF-α*) as well as *MMP-1* gene expression was dramatically downregulated in injuries treated with OEA or MEBO^®^ comparing to control injuries (Figure 10). Wounds treated with OEA had a much more significant decrease in inflammatory markers (*IL-1β* and *TNF-α*) and *MMP-1* than MEBO^®^-treated wounds. Furthermore, compared to control wounds, OEA or MEBO^®^ therapy for fourteen days resulted in significantly lower *TNF-α* and *IL-1β*, *MMP-1* relative gene expression (*p* < 0.05). Again, *TNF-α*, *IL-1β*, and *MMP-1* expression were significantly lower in OEA-treated wounds than in MEBO^®^-treated wounds.

### 3.3. In Silico Investigation

#### 3.3.1. Predicted Targets for Chemical Compounds in OEA

The inverse docking approach was used to accurately propose the most likely molecular targets of all the annotated compounds in OEA using the idTarget docking platform (http://idtarget.rcas.sinica.edu.tw) (accessed on 1 March 2022).

Divide-and-conquer docking is a unique docking strategy in which small overlapping grids are adaptively generated by idTarget to confine the examination area on the protein surfaces. This procedure allows the execution of many accurate dockings in a brief period of time. The query molecule can be virtually docked to practically all available crystal protein structures in the PDB.

A list of binding affinity scores, ranging from the most negative to the least, was created from the combined results. For each identified molecule in OEA, the best targets were determined using a threshold value of 7 kcal/mol for binding affinity. As an essential protein in the wound healing process, MMP1 was retrieved as a target protein for compounds **2**, **5**, **8**, and **12** with binding affinity scores of −9.3, −8.8, −8.1, and −12.7 kcal/mol, respectively. Several earlier studies have demonstrated that the healing of wounds is adversely affected by MMPs. Hence, using MMP inhibitors has been shown to result in outstanding outcomes in wound healing.

**Figure 11:**
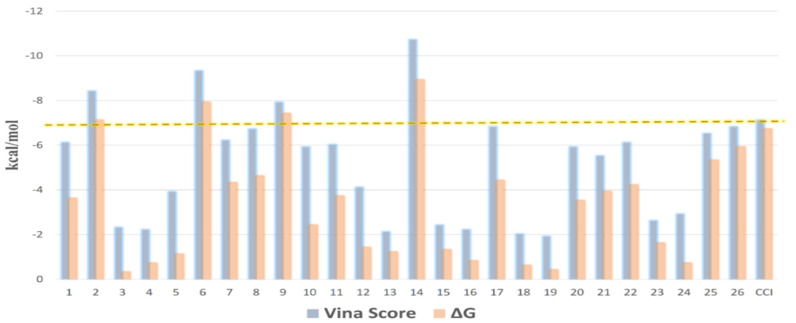
Docking scores of compounds 1–26 inside the active site of MMP1 along with their calculated Δ*G*s. The −7 kcal/mol score was set as a cutoff to select the best hits. CCl = co-crystalized ligand.

All molecules were then re-docked toward the active site of MMP1 using AutoDock Vina. It was also revealed by the Vina docking results that compounds **2**, **5**, **8**, and **12** were the structures reaching the highest-ranking scores, with binding affinity scores between −7.9 and −10.3 kcal/mol. After that, the molecular dynamic simulation-derived binding free energies (Δ*G*s) of all compounds were also calculated to further support the docking results. Once again, compounds **2**, **5**, **8**, and **12** were the top-scoring structures, with Δ*G* values between −7.1 and −8.9 kcal/mol (Figure 11).

#### 3.3.2. Binding Mode Investigation, Molecular Dynamics Simulation, and In Vitro Validation

**Figure 12:**
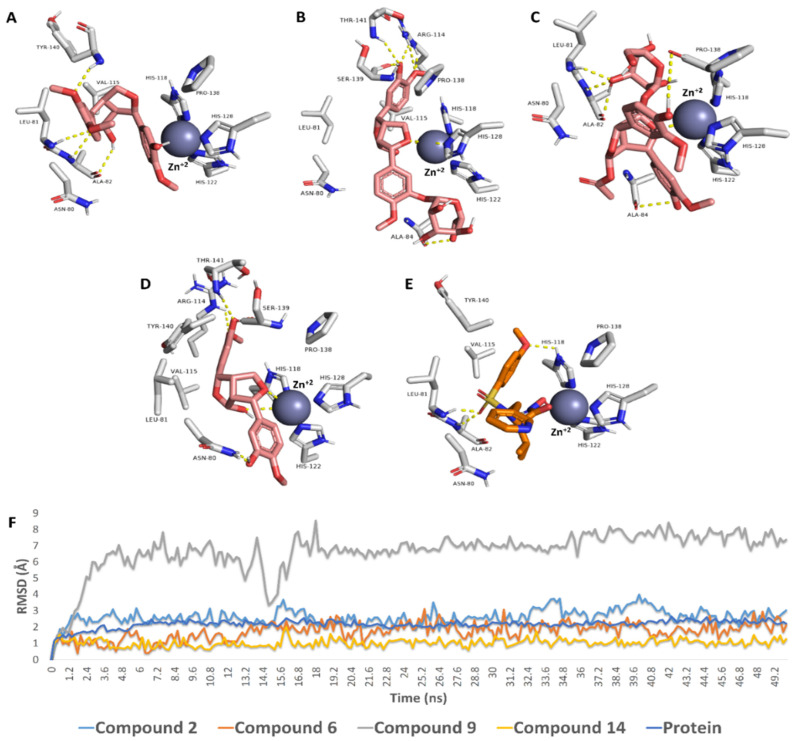
Binding poses of compounds **2**, **5**, **8**, and **12** within the potential active site of MMP1 (**A**–**E**) and that of the cocrystalized inhibitor I. The RMSDs of compounds **2**, **5**, **8**, and **12** within the potential active site of MMP1 over 50 ns of MDS (**F**).

The binding orientations of compounds **2**, **5**, **8**, and **12** (common lignans in olive; [37]) inside the binding site of MMP1 were quite similar to that of the co-crystallized ligand [59] (Figure 12). LEU-81, ALA-82, and HIS-118 are critical residues involved in hydrogen bond interactions with the co-crystalized inhibitor (Figure 12A), while VAL-115, HIS-118, and TYR-140 are critical residues implicated in hydrophobic interactions. The binding modes of compounds **2** and **8** were quite similar to that of the co-crystalized ligand, where they were H-bonded to LEU-81 and ALA-82. Moreover, they established additional H-bonds with ALA-84, PRO-138, and TYR-140 (Figure 12A,C). They also established hydrophobic interactions identical to those of the cocrystalized inhibitor. They took a slightly different orientation regarding compounds **5** and **12**. Both established a network of strong H-bonds (<2 Å) with ALA-84, ARG-114, PRO-138, SER-139, and THR-141 (Figure 12B,D). They established several hydrophobic interactions similar to those of the cocrystalized inhibitor. Additionally, they interacted with both PRO-138 and VAL-115.

We exposed the selected docked compounds to 50 ns molecular dynamic simulations (MDS) to validate these binding poses. Compounds **2**, **5**, and **12** achieved stable binding throughout the course of MDS with low deviations from their original pose (average RMSD ranged from 1 Å for compound **12** to 3 Å for compound **2**). On the other hand, compound **8** was far less stable during MDS, where it was highly fluctuating inside the binding pocket of MMP1, particularly at the beginning of the simulation until 19.2 ns, and its overall RMSD was relatively high (~6 Å).

As a validation step of the previous in silico and modeling outcomes, OEA was tested in vitro against *MMP-1*. The results showed that the OEA crude extract had *MMP-1* inhibitory activity with IC_50_ = 88 ± 0.1 µM. This potent activity could be attributed to compounds **2**, **5**, **8**, and **12** being significant components in OEA (peak area > 10,000).

## 4. Discussion

Arbequina is an olive variety that has picked up insufficient attention. Reflecting the results from the present study and the literature, different phytochemical compounds were identified from the OEA crude leaf extract, consisting of lignans, secoiridoid, triterpenes, fatty acids, megaritolactones, benzopyrene, flavonoids, and phenyl ethanoids [43]. The identified phytochemical compounds showed significant differences compared with 32 cultivars (*Bouteillan*, *Fecciaro*, *Frantoio selection*, *Manzanilla*, *Nocellara del belice*, *Picudo de Labata*, *I-79*, *Pendolino*, *O. europaea subsp. Cuspidata*, *isolate Yunnan*, *Ascolana tenera*, *Zhonglan*, *Koroneiki*, *Arbequina*, *Huaou 5*, *Nikitskii I*, *Picholine*, *Chemlal de Kabylie*, *Hojiblanca*, *Manzanilla sevillana*, *Canino*, *Cipressino*, *Rosciola*, *Nevadillo fino*, *Castellana*, *Neral*, *Olivon de Roda*, *Largueta*, *Manzanilla Greece*, *Blanqueta*, *Benizar*, *Morcona*, *and Gentile di chieti*, which are mainly collected from France, Italy, China, Spain, Greece, Azerbaijan, and Algeria), especially in the lignans and secoiridoid content, which are predominant in OEA crude extract [60]. The major class of phenolics (i.e., flavonoids, iridoids, triterpenes, and fatty acids) displayed the same profiles as those reported in the literature. However, the phenolic profiles of olive leaves previously detected in several common cultivars were marginally distinct from those found in this work [61].

Wound healing is a network-dynamic procedure of bringing back tissue construction in damaged tissues close to its probable ordinary state [62]. This requires three phases: (1) an inflammatory stage that involves a section of proinflammatory mediators and suppression of the immune system, (2) a proliferative stage in which fibroblast proliferation, collagen aggregation, and new blood vessel formation occurs, and (3) a remodeling stage that involves wounded tissue regeneration and reconstruction [63,64]. For efficient treatment, remedies that hasten wound healing with potential involvement in all process phases, fewer side effects, and limited cost are needed.

In this work, a significant contraction in the wounded area of excision wounds of experimental animals was revealed compared to the control wounds in topical OEA treatment. These observations are due to the accelerated wound contraction rate in OEA-treated animals. To speed up and encourage wound closure, wound contraction is the centripetal movement of the wound’s margins [65,66,67]. Wound contraction therefore indicates re-epithelialization, keratinocyte differentiation, granulation, fibroblast proliferation, and proliferation [67]. The obtained results agree with other works [68,69,70,71].

Complex interactions between cells and numerous growth factors are required during wound healing processes [72]. Throughout the phases of wound healing, *TGF-β* has an extremely urgent role in the inflammation and hemostasis stage. Inflammatory cells like neutrophils and macrophages are attracted to and stimulated by *TGF-β*. At the same time, multiple cellular activities, such as angiogenesis, re-epithelialization, granulation tissue growth, and ECM deposition, are orchestrated by it during the proliferative phase [72]. Fibroblasts are encouraged to multiply and diversify into myofibroblasts that participate in the remodeling stage of wound contraction [73]. Pastar et al. and Haroon et al. argued that chronic wounds usually exhibit diminished *TGF-β1* signaling [74,75]. Feinberg et al. clarified that *TGF-β1* attenuates the expression of collagenases, weakening collagen and ECM [76]. These notes are consistent with our observations, which concluded that enhanced *TGF-β1* expression following OEA topical application hastens wound healing, as noticed by the gene expression and the accelerated wound healing in this study (Figure 9).

Proinflammatory cytokines like IL-1*β* and TNF-*α* are only important during the initial response of skin wound healing in the inflammatory phase. Adequate TNF-*α* and IL-1*β* expression is required for neutrophil recruitment and the cleanup of the wound area to get rid of bacteria and other contaminants. Additionally, these inflammatory mediators are considered potent MMP inducer in fibroblasts and inflammatory cells. The damaged ECM is degraded and eliminated by MMP to facilitate wound repair [77]. Nevertheless, prolonged inflammation stage results in a deformative healing process, as proteinases and cytokines damage the tissue, resulting in the progress of chronic wounds. *TNF-α* is a crucial proinflammatory cytokines secreted by macrophages, which, along with *IL-1β*, hinders collagen production and fibroblast proliferation [78]. *TNF-α* activates NF-κB, which in round activates a multitude of proinflammatory cytokines encompassing proteases, such as MMP and *TNF-α* itself, to give rise to free soluble *TNF-α*. As a result, these inflammatory cytokine effects are potentiated [79]. Thus, suppressing *TNF-α* and *IL-1β* by OEA could suppress prolonged inflammation and avoid defective wound healing (Figure 10). These investigations made us assume that OEA quickens and improves the curing process.

Conversely, area, collagen disruption, and hence ECM destruction could be stimulated by reactive oxygen species at high levels in the wounded area. Events like angiogenesis and re-epithelization, which are crucial for wound healing, are reduced when the ECM is damaged [80,81]. Furthermore, inflammation can be induced by elevated ROS, increasing proinflammatory cytokines, and as result lengthening inflammation [82]. Due to its SOD activity and H_2_O_2_ scavenging activity, OEA may have antioxidant properties. This was confirmed by in vitro antioxidant studies, potentially eliminating ROS, and enhancing wound-healing properties. The phenolic contents of OEA are responsible for these antioxidant actions [43].

Additionally, persistent inflammation results in wound healing failure, and wounds typically start a pathological state that requires more aggressive treatments. The capability of an agent to deal with the inflammatory wound process will either improve or quicken the healing process [83]. As opposed to that, MMPs have been proven to have a crucial impact in wound healing, and their inhibition has been associated with significant positive outcomes. The ability of some lignan-related compounds (**2**, **5**, **8**, and **12**) to interact with *MMP-1* was revealed by an in silico investigation of the OEA primary metabolites. As a validation step, OEA was tested in vitro against *MMP-1*. The results showed that the OEA crude extract had *MMP-1* inhibitory activity with IC_50_ = 88 ± 0.1.

## 5. Conclusions

As presented here, the Arbequine olive leaves extracts, have a different phytochemical composition. Through an increased rate of wound closure, enhancement of TGF-1, and suppression of inflammatory markers (TNF- and IL-1), this extract demonstrated outstanding wound healing activity. The ability of some lignan-related molecules (**2**, **5**, **8**, and **12**) to interact with *MMP-1* was suggested by a series of virtual screening and physics-based experiments, which were conducted to explore the binding modes of molecules within the potential active site of each molecular target. This study reflected the potential of Arbequina cultivars as a source of polyphenolic wound healers, like wild cultivars.

## Figures and Tables

**Table 1:** Primers used for real-time PCR.

Name of Gene	Accession Number		Primer Sequence
*IL-1β*	*NC_013670.1*	forward	5′-AGCTTCTCCAGAGCCACAAC-3′
		reverse	5′-CCTGACTACCCTCACGCACC-3′
*GAPDH*	*NC_013676.1*	forward	5′-GTCAAGGCTGAGAACGGGAA-3′
		reverse	5′-ACAAGAGAGTTGGCTGGGTG-3′
*TGF-β*	*NC_013672.1*	forward	5′-GACTGTGCGTTTTGGGTTCC-3′
		reverse	5′-CCTGGGCTCCTCCTAGAGTT-3′
*TNF-α*	*NC_013680.1*	forward	5′-GAGAACCCCACG GCTAGATG-3′
		Reverse	5′-TTCTCCAACTGGAAGACGCC-3′
*MMP-1*		forward	5′-TTTCCCCCTGGCGCCGGCGTT-3′
		Reverse	5′-CTCGTGCGCTGCCACCAGG-3′

**Table 2:** Dereplicated metabolites from LC-HRESIMS analysis of *OEA* leaves crude extract.

Metabolite No.	Source	MF	RT (min)	*m/z*
**1**	*OEA*	C_20_H_22_O_7_	2.01432	375.1444
**2**	*OEA*	C_21_H_24_O_7_	2.01740	389.1600
**3**	*OEA*	C_11_H_12_O_5_	2.20205	223.06055
**4**	*OEA*	C_15_H_16_O_9_	2.37151	341.08660
**5**	*OEA*	C_26_H_32_O_12_	2.40519	537.1972
**6**	*OEA*	C_22_H_36_O_13_	2.49208	509.22142
**7**	*OEA*	C_27_H_34_O_13_	2.53020	567.2078
**8**	*OEA*	C_28_H_34_O_13_	2.75095	579.20783
**9**	*OEA*	C_26_H_34_O_13_	3.02300	555.2078
**10**	*OEA*	C_22_H_24_O_8_	3.23649	417.15389
**11**	*OEA*	C_8_H_10_O_3_	3.78010	155.0708
**12**	*OEA*	C_15_H_16_O_9_	3.78010	341.0873
**13**	*OEA*	C_18_H_36_O_5_	4.09611	331.24803
**14**	*OEA*	C_11_H_14_O_6_	4.18760	243.0869
**15**	*OEA*	C_30_H_48_O_4_	5.96396	473.36213
**16**	*OEA*	C_21_H_42_O_2_	6.01750	327.3263
**17**	*OEA*	C_22_H_36_O_13_	9.11727	509.2235
**18**	*OEA*	C_26_H_38_O_13_	10.5214	557.2229
**19**	*OEA*	C_15_H_15_O_3_	12.8056	243.1013
**20**	*OEA*	C_18_H_35_O_5_	14.6147	331.2490
**21**	*OEA*	C_18_H_32_O_3_	21.7898	295.2280
**22**	*OEA*	C_30_H_50_O_2_	29.2663	443.3880
**23**	*OEA*	C_30_H_49_O_3_	30.5872	457.3682

## Data Availability

The data presented in this study are available in article and supplementary material.

## Supplementary Materials

The following supporting information can be downloaded at: https://www.mdpi.com/article/10.3390/metabo12090791/s1, Supplementary file. References [84,85,86,87] are cited in the supplementary materials.
