# Supplementary material for: The Wound-Healing Potential of Olea europaea L. Cv. Arbequina Leaves Extract: An Integrated In Vitro, In Silico, and In Vivo Investigation
Source: Metabolites. 2022 Aug 25;12(9):791. doi: 10.3390/metabo12090791 (PMC9503157; doi:10.3390/metabo12090791)
Supplement: Supplementary file 1 [file metabolites-12-00791-s001.zip › metabolites-1814821-supplementary.pdf]

Supplementary Material File

The Wound-Healing Potential of *Olea europaea* L. Cv. Arbequina Leaves Extract: An Integrated In Vitro, In Silico, and In Vivo Investigation

Tarfah Al-Warhi [1], Abeer H. Elmaidomy [2], Sherif A. Maher [3], Dalia H. Abu-Baih [3], Samy Selim [4], Mha Albqmi [5], Mohammad M. Al-Sanea [6,*], Taghreed S. Alnusaire [7], Mohammed M. Ghoneim [8,9], Ehab M. Mostafa [8,10], Shaimaa Hussein [11], Ashraf K. El-Damasy [12,13], Entesar Ali Saber [14,15], Mahmoud A. Elrehany [3], Ahmed M. Sayed [16,*], Eman M. Othman [17,18], Mohamed El-Sherbiny [19,20] and Usama Ramadan Abdelmohsen [21,22,*]

[1] Department of Chemistry, College of Science, Princess Nourah bint Abdulrahman University, Riyadh 11671, Saudi Arabia
[2] Department of Pharmacognosy, Faculty of Pharmacy, Beni-Suef University, Beni-Suef 62511, Egypt
[3] Department of Biochemistry, Faculty of Pharmacy, Deraya University, New Minia 61111, Egypt
[4] Department of Clinical Laboratory Sciences, College of Applied Medical Sciences, Jouf University, Sakaka 72341, Saudi Arabia
[5] Chemistry Department, College of Science and Arts, Jouf University, Alqurayyat 77447, Saudi Arabia
[6] Pharmaceutical Chemistry Department, College of Pharmacy, Jouf University, Sakaka 72341, Saudi Arabia
[7] Biology Department, College of Science, Jouf University, Sakaka 72341, Saudi Arabia
[8] Pharmacognosy and Medicinal Plants Department, Faculty of Pharmacy (Boys), Al-Azhar University, Cairo 11884, Egypt
[9] Department of Pharmacy Practice, College of Pharmacy, Al Maarefa University, Ad Diriyah 13713, Saudi Arabia
[10] Department of Pharmacognosy, College of Pharmacy, Jouf University, Sakaka 72341, Saudi Arabia
[11] Department of Pharmacology, College of Pharmacy, Jouf University, Sakaka 72341, Saudi Arabia
[12] Department of Medicinal Chemistry, Faculty of Pharmacy, Mansoura University, Mansoura 35516, Egypt
[13] Brain Science Institute, Korea Institute of Science and Technology (KIST), Seoul 02792, Korea
[14] Department of Histology and Cell Biology, Faculty of Medicine, Minia University, Minia 61519, Egypt
[15] Department of Histology and Cell Biology, Deraya University, Universities Zone, New Minia 61111, Egypt
[16] Department of Pharmacognosy, Faculty of Pharmacy, Nahda University, Beni-Suef 62513, Egypt
[17] Department of Biochemistry, Faculty of Pharmacy, Minia University, Minia 61519, Egypt
[18] Department of Bioinformatics, Biocenter, University of Würzburg, Am Hubland, 97074 Wuerzburg, Germany
[19] Department of Basic Medical Sciences, College of Medicine, AlMaarefa University, Riyadh 11597, Saudi Arabia
[20] Department of Anatomy, Faculty of Medicine, Mansoura University, Mansoura 35516, Egypt
[21] Department of Pharmacognosy, Faculty of Pharmacy, Minia University, Minia 61519, Egypt
[22] Department of Pharmacognosy, Faculty of Pharmacy, Deraya University, New Minia 61111, Egypt
* Correspondence: mmalsanea@ju.edu.sa (M.M.A.-S.); ahmedpharma8530@gmail.com (A.M.S.); usama.ramadan@mu.edu.eg (U.R.A.)

Abstract: *Olea europaea* L. Cv. Arbequina (OEA) (Oleaceae) is an olive variety species that has **Abstract:** *Olea europaea* L. Cv. Arbequina (OEA) (Oleaceae) is an olive variety species that has received little attention. Besides our previous work for the chemical profiling of OEA leaves using LC–HRESIMS, an additional 23 compounds are identified. An excision wound model is used to measure wound healing action. Wounds are provided with OEA (2% *w*/*v*) or MEBO® cream (marketed treatment). The wound closure rate related to vehicle-treated wounds is significantly increased by OEA. Comparing to vehicle wound tissues, significant levels of *TGF-β* in OEA and MEBO® ($p < 0.05$) are displayed by gene expression patterns, with the most significant levels in OEA-treated wounds. Proinflammatory *TNF-α* and *IL-1β* levels are substantially reduced in OEA-treated wounds. The capability of several lignan-related compounds to interact with *MMP-1* is revealed by extensive in silico investigation of the major OEA compounds (i.e., inverse docking, molecular dynamics simulation, and Δ*G* calculation), and their role in the wound-healing process is also characterized. The potential of OEA as a potent *MMP-1* inhibitor is shown in subsequent in vitro testing (IC_{50} = 88.0 ± 0.1 nM). In conclusion, OEA is introduced as an interesting therapeutic candidate that can effectively manage wound healing because of its anti-inflammatory and antioxidant properties.

Keywords: *Olea*; olive; LC–HRESIMS; wound; *TNF-α*; *TGF-β*; *MMP-1*; virtual docking

Methods

LC.MS metabolomics analysis

The crude ethanolic extract from OEA was adjusted to 1 mg/mL for the mass spectrometry analysis. The recovered ethanolic extract was subjected to metabolic analysis using LC-HRESIMS according to Elmaidomy and coworkers 2020 [1]. An Acquity Ultra Performance Liquid Chromatography system connected to a Synapt G2 HDMS quadrupole time-of-flight hybrid mass spectrometer (Waters, Milford, USA) was used. Positive and negative ESI ionization modes were utilized to carry out the high-resolution mass spectrometry coupled with a spray voltage set at 4.5 kV, with the capillary temperature set at 320 °C, and mass range from *m/z* 150–1500. The MS dataset was processed, and data were extracted using MZmine 2.20 based on the established parameters. Mass ion peaks were detected and accompanied by chromatogram builder and chromatogram deconvolution (specify the program used for this chromatogram building and deconvolution). The local minimum search algorithm was addressed, and isotopes were also distinguished via the isotopic peaks of grouper. Missing peaks were displayed using the gap-filling peak finder. An adduct search along with a complex search was carried out. The processed data set was next subjected to molecular formula prediction and peak identification (specify the program used). The positive and negative ionization mode data sets from the respective extract were dereplicated against the DNP (Dictionary of Natural Products) databases (specify the databases used).

Docking Molecular Dynamic Simulation and Binding Free Energy Calculation

Desmond v. 2.2 software, the MDS machine of Maestro-Schrodinger platform, was used for performing MDS experiments. This software applies the OPLS force field [2]. Protein systems were constructed using the System Builder option, where the protein structure was checked for any missing hydrogen, the protonation state of the amino acid residues was set at pH = 7.4, and the native co-crystalized water molecules were removed. Thereafter, the whole structure was embedded in an orthorhombic box of modeled water molecules together with 0.15 M Na+ and Cl− ions in 20 Å^3 solvent buffer. Subsequently, the prepared systems were energy minimized and equilibrated for 10 ns. For protein-ligand complexes, the top-scoring poses were papered and utilized as a starting point for the simulations. Desmond software automatically parameterizes inputted ligands structures during the system building step according to the OPLS force field. For NAMD-performed simulations [3], the protein structures were built and optimized by using the QwikMD toolkit of the VMD software 1.9.3 [4]. The parameters and topologies of the tested compounds were generated using the Charmm27 force field with the online software Ligand Reader and Modeler (http://www.charmm-gui. org/?doc=input/ligandrm). Afterward, these parameters and topology files were loaded to VMD to readily read the protein–ligand complexes without errors and then conduct the simulation step.

Binding Free Energy Calculations

Absolute binding free energy calculations (Δ*G*) were estimated using the free energy perturbation (FEP) method [4]. According to the previously reported method by Kim and coworkers, this method calculates the binding free energy $\Delta G_{binding}$ following the equation: $\Delta G_{binding} = \Delta G_{Complex} - \Delta G_{Ligand}$. The value of each Δ*G* is estimated from a separate number of simulations runs using NAMD software. All input files needed for the simulation by NAMD can be generated by using the online website Charmm-GUI (https://charmm-gui.org/?doc=input/afes.abinding). Thereafter, we can use these generated files in NAMD to produce the required simulations using the FEP calculation function in NAMD software. A 5 ns long equilibration was achieved in the NPT ensemble at 300 K and 1 atm (1.01325 bar) with Langevin piston pressure (for "Complex" and "Ligand") in the presence of modeled water molecules. Then, 10 ns FEP simulations were carried out for each tested compound, and the last 5 ns of the free energy values were measured for the final

free energy values [5]. Finally, the generated trajectories were visualized and analyzed using VMD software 1.9.3 [4] (add reference for the software and version used).

References

1. Elmaidomy, A. H., Alhadrami, H. A., Amin, E., Aly, H. F., Othman, A. M., Rateb, M. E., Hetta, M. H., Abdelmohsen, U. R. and M Hassan, H. Anti-inflammatory and antioxidant activities of terpene-and polyphenol-rich *Premna odorata* leaves on alcohol-inflamed female wistar albino rat liver. *Molecules.* 2020; 25**:** 3116.
2. Chow, E., Rendleman, C. A., Bowers, K. J., Dror, R. O., Hughes, D. H., Gullingsrud, J., Shaw, D. E. (2008). Desmond performance on a cluster of multicore processors. *DE Shaw Research Technical Report DESRES/TR--2008-01*.
3. Phillips, J. C., Braun, R., Wang, W., Gumbart, J., Tajkhorshid, E., Villa, E., ... and Schulten, K. Scalable molecular dynamics with NAMD. *J. Comput. Chem.* 2005; *26*(16): 1781-1802.
4. Humphrey, W., Dalke, A., & Schulten, K. (1996). VMD: visual molecular dynamics. *Journal of molecular graphics*, *14*(1), 33-38.
5. Ngo, S. T., Tam, N. M., Pham, M. Q., and Nguyen, T. H. Benchmark of popular free energy approaches revealing the inhibitors binding to SARS-CoV-2 mpro. *J. Chem. Inf. Model*. 2021; *61*(5): 2302-2312.
